# Comparison of Male and Female Patients in Louisiana Medical Marijuana Dispensaries

**DOI:** 10.3390/jcm9061865

**Published:** 2020-06-15

**Authors:** H. Raymond Allen, Doug Boudreaux, Jeffrey N. Keller

**Affiliations:** 1 Pennington Biomedical Research Center, 6400 Perkins Road, Baton Rouge, LA 70820, USA; Ray.Allen@pbrc.edu; 2 President of Louisiana Association of Therapeutic Alternatives, Hope Pharmacy, 1410 Kings Hwy, Shreveport, LA 71103, USA; doug@boudreauxscompounding.com; 3 Keller-Lamar Health Foundation, 5321 Corporate Boulevard, Baton Rouge, LA 70808, USA

**Keywords:** demographics, dispensary, gender, medical marijuana, pharmacist, web-based

## Abstract

Relatively little is known in terms of patient demographics, indications, previous cannabis use, or the forms and dosages of medical marijuana (MM) dispensed for patients at MM dispensaries. Even less is known in terms of how male and female patients may differ in each of these aspects. The goal of the current study was to examine each of these variables using a retrospective analysis of deidentified patient data from MM dispensaries in Louisiana. Deidentified data were analyzed from web-based pharmacist–patient consultations at MM dispensaries throughout Louisiana. Data were collected during the first 6 months following the initiation of the MM dispensing program in Louisiana. A total of 1195 MM patients (598 male/597 female) were included in the analyses. The average age of the sample was 51.9 years (±14.8) and it was composed primarily of white patients (86.7%). Males and females were nearly identical in terms of average age, race, previous cannabis use, indication profile, and MM recommendations. Differences between males and females were observed in terms of opioid use, history of psychosis, presence of more than one indication, and the duration of previous cannabis use. Our data indicate that, in MM dispensaries of the Deep South state, there are numerous similarities—and some potentially important differences—between male and female MM patients. The importance of these differences, and the importance of continued data collection/analysis, for improving MM dispensing are discussed.

## 1. Introduction

Recent surveys suggest that around 8% of adults in the United States use marijuana, with upwards of 4% of those surveyed reporting the daily use of marijuana [1,2]. National surveys have found that 50% of individuals with medical conditions, such as stroke, diabetes, renal disease, and cancer, have reported marijuana use in the last 12 months [3]. Currently, 30 states and the District of Columbia have approved medical marijuana (MM) use, with MM believed to represent approximately 10% of adult marijuana users [4]. There is considerable variability in the state-approved MM programs in terms of the indications for which MM has been approved, with very little guidance currently available for physicians to use for determinations of the optimal dose and form of MM to recommend to a patient. Physicians repeatedly cite that they lack the education, training, and evidence-based guidelines necessary for discussing marijuana and making recommendations for MM [5,6].

Despite the growing number of state-approved MM programs, relatively little is known in terms of even basic patient demographics for individuals participating in MM programs [7,8]. Similarly, little is currently known in terms of the history of previous cannabis use in MM patients and the profile of MM recommendations within an individual state. Lastly, our understanding of how male and female MM patients may potentially differ in each of these different aspects of MM dispensing remains to be elucidated. Developing a more uniform and systematic way of collecting patient and recommendation data as part of a MM patient consultation could facilitate the development of future evidence-based guidelines focused on the dispensing of MM.

In 2016, the Louisiana governor signed Senate Bill (SB) 271 and SB 180, which allowed Louisiana physicians to recommend MM for a limited number of indications and for patients and caregivers to possess MM. In 2018, the governor signed HB579 and HB627, which expanded the range of indications to autism, post-traumatic stress disorder (PTSD), intractable pain, cancer, human immunodeficiency virus infection/acquired immune deficiency syndrome (HIV/AIDS), cachexia, seizure disorders, spasticity, Crohn’s disease, muscular dystrophy, Parkinson’s disease (PD), multiple sclerosis (MS), and some forms of autism. Currently, nine MM dispensaries are in operation in Louisiana, with one dispensary established by the Louisiana Department of Health (LDH) in each of the nine regions of Louisiana. At the present time, MM in the State of Louisiana must be dispensed by a pharmacist and is currently dispensed as either a tetrahydrocannabinol (THC)-rich, a cannabidiol (CBD)-rich, or a balanced formulation with dosage recommendations ranging from 0.2 to 10 mg.

This manuscript describes a retrospective analysis of patient demographics, history of cannabis use, indications, and recommendations collected during the first 6 months of the Louisiana MM program. The focus of the study was to identify the similarities and differences between male and female MM patients in each of these aspects as related to MM dispensing. These data were collected as part of a web-based pharmacist–patient consultation program provided to the nine MM dispensaries throughout Louisiana.

## 2. Materials and Methods

### 2.1. Medical Marijuana Dispensaries

The deidentified data collected for this analysis were obtained as part of a web-based platform used as part of an ongoing database for pharmacist–patient consults initiated by the Louisiana Association of Therapeutic Alternatives (LATA). At the time of this study, all nine MM dispensaries in Louisiana participated in this web-based effort (The Apothecary Shoppe, Lafayette, LA, USA; Capitol Wellness Solutions, Baton Rouge, LA, USA; Delta Medmar, West Monroe, LA, USA; Greenleaf Dispensary, Bayou Cane, LA, USA; Hope Pharmacy, Shreveport, LA, USA; H & W Drug Store, New Orleans, LA, USA; The Medicine Cabinet Pharmacy, Madisonville, LA, USA; The Medicine Cabinet, Alexandria, LA, USA; Willow Pharmacy Inc, Madisonville, LA, USA). All patients in the analyses were seen by community physicians, with an approved existing indication required for the recommendation of MM.

Each participating dispensary was equipped with access to the web-based application on a desktop that was used by each pharmacist for the patient consult. Data for this initial feasibility effort were collected during the first 6 months of operation for MM in the State of Louisiana (August 2019–Feb 2020). Pharmacists could use the web-based platform or alternative methods in order to obtain the state-mandated consultation between pharmacists and patients in the MM dispensary. This retrospective study used deidentified data with no patient identifiers. Permission was obtained from all MM dispensaries and LATA. Data were used from patients who indicated during the pharmacist–patient consultation that they approved of having their data used for research purposes.

### 2.2. Web-Based Platform

The State of Louisiana requires that MM be dispensed by a state-approved pharmacist. Each consultation was performed by the pharmacist located in each state-licensed dispensary using the LATA web-based platform or an alternative mode of data collection. Each pharmacist was required to log onto the LATA website with their personal code and use the secure link to gain access to the web-based application. The questions on the web-based platform were used to guide, and provide documentation of, the pharmacist-led patient consultation. Patients were asked about specific medications (opioids, blood thinners, anti-seizure medications), basic demographics, and previous cannabis use. Questions on previous cannabis use included the modality, frequency, and duration of use. The state-approved indication(s), providing the basis for the recommendation was recorded. Information regarding the form, route, dosage, and frequency of MM was recorded for each patient. Documentation confirming that the patient understood the HIPAA, as well as the potential implications of federal/state laws and potential employer rules, were also captured. In order to determine the feasibility of using the web-based platform as a potential means to build a research registry, the pharmacists also asked and recorded whether patients were willing to have their data used for research purposes. The web-based platform was secured through Transformyx LLC (Baton Rouge, LA) and developed and maintained by the not for profit Keller-Lamar Health Foundation for the purposes of this LATA focused effort.

## 3. Results

### 3.1. Patient Demographics

The study sample was composed of 1195 patients with a nearly identical distribution of male (598) and female (597) patients (Table 1). The average age of the study sample was 51.9 years (±14.8) and comprised primarily of white (86.7%) and black patients (11.5%) (Table 1). The average ages of male (52.2 years ± 14.7) and female (51.2 years ± 14.8) patients were not significantly different (Table 1). The percentages of white males (85.2%) and white females (86.9%), as well as black males (11.5%) and black females (11.1%), were nearly identical (Table 1). Males were nearly three times as likely to be currently taking opioids (27.1%) as compared to females (9.2%) (Table 1). Female patients were nearly 20 times more likely to have a history of psychosis (21.4%) as compared to males (1.3%) (Table 1).

### 3.2. Previous Cannabis Use

Patients were asked during the consultation to volunteer information on their previous cannabis use (not required to answer) with 97.3% of males and 96.8% of females providing a response (Table 2). The majority of patients reported some form of previous cannabis use (64.3%), with similar responses between males (68.3%) and females (60.3%) (Table 2). The majority of males reported 5 or more years of previous cannabis use (56.1%), while the majority of females reported less than 5 years of previous cannabis use (61.5%) (Table 2). The 1160 patients that reported using cannabis previously were also asked to self-report the modality of cannabis used (not required) and a total of 1013 responses were collected (Table 3). The version of the web-based platform used in the current study did not collect information regarding whether previous cannabis use was recreational or medical. Males (56%) and females (50.8%) reported a similar rate of smoking cannabis previously (Table 3). Males were twice as likely (11.1%) as females (5.1%) to use THC oil (Table 3), and females were twice as likely to consume edibles (25.7%) as compared to males (12.4%) (Table 3).

### 3.3. Indications for MM Recommendations

A total of 1422 indications were reported as the basis for the MM recommendation in the 1195 patients (Table 4). Females were nearly three times as likely (144 instances) as compared to males (51 instances) to have more than one indication reported as the basis for their MM recommendation (Table 4). The top four indications reported for males and females were nearly identical: intractable pain (46.4%/53.1%), PTSD (18.9%/12.3%), severe muscle spasms (10.5%/12.3%) and cancer (7.3%/9.3%) (Table 4).

### 3.4. MM Recommendations

The THC-rich formulation was the most frequent MM recommendation (52.3%), followed by the balanced formulation (41.4%) and CBD-rich formulation (6.3%) (Table 5). No apparent significant differences were observed between males and females in terms of the rates at which THC-rich, balanced, and CBD-rich formulations were recommended (Table 5). Similarly, there did not appear to be significant differences between males and females in terms of the doses of THC-rich, balanced, and CBD-rich formulations recommended (Table 5).

## 4. Discussion

Currently, only a small number of publications exist on the topics related to MM dispensaries, and nearly all of these existing studies have focused on patients with specific indications [9,10], comparisons of younger and older MM patients [11,12], perceptions and training related to dispensary staff [13,14], or associations between the MM dispensary and different aspects of the local community [15,16]. Our current study adds to this existing literature by reporting on multiple characteristics within a broad spectrum of MM patients, and to the best of our knowledge is potentially the first study to examine the similarities and differences between male and female MM patients.

We identified that there were many more similarities than differences between male and female MM patients including a nearly identical representation of age, race, indications, and MM recommendations. In the current study, 50% of the patients were male and 50% were female, which is in contrast to the majority of previous publications, which have described a higher percentage of male MM patients. Similarly, the average age of 51.9 years for MM patients in the current study is lower than has been reported in several previous studies. While the basis for these differences between the patient sample in this study and previous publications is not known, it may be related to the observation that these deidentified data were collected during the initial 6 months of the launch of the MM program in Louisiana and, as such, represent a certain amount of “pent-up demand” within the state. It will be important to determine the stability of these two observations, as the number of physicians and patients involved in the MM program continues to grow.

One of the key differences we observed between male and female MM patients was in regard to the presence of potentially contraindicated medications and medical conditions. For example, we observed that males were much more likely than females to currently be using opioids, and male MM patients reported having used cannabis products previously for five years or longer at a significantly higher rate than female MM patients. Currently, there is tremendous interest in elucidating the potential for MM use to potentially decrease the use of opioids in some medical conditions and, conversely, the potential for MM to potentially exacerbate opioid abuse and the negative effects of opioid use in some patients [17,18,19,20]. The majority of opioid-related studies to date have focused on the interplay between opioid use and recreational marijuana use and have not specifically examined marijuana use as part of MM programs. Our current data suggest that developing a better understanding of the potential interplay between opioids and MM patient outcomes will have to take into account potentially important gender differences in terms of both opioid use and the profile of previous cannabis use outside of the MM setting.

We identified an elevated incidence of a self-reported history of psychosis in female MM patients in the current study, as compared to male MM patients. Female MM patients were also more likely to have more than one indication reported as the basis for their MM recommendation. Interestingly, the rank order frequency of the different indications was nearly identical between males and females, as were the forms and doses of MM recommended by their physicians. In future studies, it will be important to not only elucidate whether psychosis is elevated in female MM patients in a larger multistate sample, but to also define the prevalence of the different types of psychosis within male and female MM patients. While the basis for the increased percentage of female patients with more than one indication is not known, it will be important to elucidate whether this is due to potential gender differences in patient–physician interactions. For example, previous studies have shown that females are more likely to discuss symptoms and medical conditions with their physician as compared to males [21]. Identifying the potential for gender-based under- or over-reporting of indications as related to MM is crucial for the success of MM outcome studies in the future [22,23].

Previous reports have suggested both a potentially beneficial effect, as well as a potentially negative effect [23,24,25,26,27,28,29,30], for cannabis use in the context of human disease. The food and drug administration (FDA) has approved a number of cannabis-related therapies, including synthetic THC compounds, as well as plant-derived CBD [23,24,25,26]. In addition to defining the potential for MM to produce beneficial and/or adverse outcomes for specific indications in different patient profiles, it will be equally important to understand the specifics surrounding the MM product itself. For example, different methodologies for extraction/purification, as well as the characteristics of the plant biomass used for extractions (growth conditions, plant genetics, etc.), will undoubtedly contribute to the outcome of MM on patient health and, therefore, need to be linked on the web-based platform to patient outcomes. Similarly, it will be important to obtain an accurate profile of the total cannabis intake (medical and non-medical) for MM patients based on the potential for entourage effects [23,24,25,26,27,28], endocannabinoid signaling, and non-cannabinoid signaling in mediating observed effects in patients.

There are considerable barriers for patients to access MM as a potential therapeutic option, including out-of-pocket expenses, difficulties in finding a physician willing to make an MM recommendation, lack of education on the topic, and social stigma. Currently, we do not know if males and females potentially differ in terms of these obstacles, or potentially differ in response to other potential barriers to MM use. Web-based platforms, including the platform used in the current study, may provide an economical and feasible method for the collection of these and additional MM-related patient data in the future. It will be important in the future to use electronic health record platforms that are focused on the multiple unique aspects associated with MM dispensing (including the platform outlined in the current study) in order to securely and seamlessly capture outcomes and adverse events for MM patients. Community physicians incorporating MM into their practice often rely on self-initiated, non-secure, fragmented, and less-than-optimal methodologies to monitor their MM patients. In addition to increasing the risk of a potential data breach and compromised patient confidentiality, these physician-initiated efforts have limited efficacy in patient monitoring.

The current study had several weaknesses, including a large dependence upon patient self-report data, and the voluntary nature of the study where pharmacists were able to use alternative methods for completing the state-mandated pharmacist consultation with MM patients. In addition, some questions were voluntary (i.e., previous history, duration, and type of cannabis use), broad in scope (i.e., previous history of psychosis), and lacked potentially important follow-up questions (i.e., was previous cannabis use recreational or medical?). Revisions to the current web-based platform, as well as the design of future web-based platforms, should attempt to minimize the potential deleterious effects of these and related issues. Lastly, the deidentified data were collected during the first 6 months of the initiation of the MM program in Louisiana, with only three forms of MM available to patients (THC-rich, CBD-rich, and balanced formulations) in tincture form only.

In conclusion, our data indicate that during the first 6 months of the Louisiana MM program male and female MM patients exhibited numerous similarities and some potentially important differences. In particular, gender differences were observed in terms of current opioid use, history of psychosis, history of cannabis use, and presence of more than one indication. In future studies, it will be important to develop a better understanding of the reproducibility of these findings in larger patient samples from multiple states and to determine the potential impact of these observed findings in MM outcome and evidence-based studies.

## Figures and Tables

**Table 1:** Comparison of male and female patients in Louisiana MM dispensaries.

Demographics	Total Sample	Male	Female
Characteristic, N (%)	1195	598 (50)	597 (50)
Age, mean (SD)	51.9 (14.8)	52.2 (14.7)	51.2 (14.8)
White	1035 (86.7)	510 (85.2)	519 (86.9)
Black	138 (11.5)	72 (12.0)	66 (11.1)
Mexican Indian or Alaskan native	15 (1.3)	7 (1.2)	8 (1.3)
Asian	5 (0.4)	2 (0.3)	3 (0.5)
Native Hawaiian	2 (0.2)	1 (0.2)	1 (0.2)
History of psychosis	136 (11.3)	8 (1.3)	128 (21.4)
Currently taking opioids	217 (18.2)	162 (27.1)	55 (9.2)
Currently taking antiseizure medicine	56 (4.6)	23 (3.8)	32 (5.4)
Currently taking blood thinner	6 (0.4)	3 (0.5)	3 (0.5)

**Table 2:** Comparison of male and female self-reports for previous cannabis use.

Previous Cannabis Use	Total	Men	Women
Responses, N (%)	1160	582 (50)	578 (50)
No Previous Cannabis use	415 (35.7)	185 (31.7)	230 (39.7)
Yes to Previous Cannabis use	745 (64.3)	397 (25.9)	348 (60.3)
* Less than 1 year of previous use	234 (20.1)	103 (17.9)	131 (37.6)
* 1–4 years previous use	154 (13.2)	71 (17.9)	83 (23.9)
* 5–9 years previous use	115 (9.9)	74 (18.6)	41 (11.8)
* 10 or more years previous use	242 (20.8)	149 (37.5)	93 (26.7)

* the length of time of the previous exposure to cannabis (as opposed to yes or no to having previous history of cannabis use).

**Table 3:** Comparison of male and female self-reports of types of previous cannabis use.

Type of Previous Use	Total Sample	Male	Female
Reported cannabis use N (%)	1013	466	547
Smoked	539 (53.2)	261 (56)	278 (50.8)
Edibles	199 (19.6)	58 (12.4)	141 (25.7)
CBD oil	157 (15.4)	75 (16.1)	82 (15.0)
THC oil	80 (7.8)	52 (11.1)	28 (5.1)
Cream	38 (3.7)	20 (4.3)	18 (3.3)

**Table 4:** Comparison of indications used for MM recommendations in male and female patients in Louisiana MM dispensaries.

Types of Indications	Total Sample	Male	Female
Indication, N (%)	1422	657 (46.2)	765 (53.8)
Autism	6 (<0.1)	3 (0.4)	3 (0.4)
Cachexia	13 (<0.1)	6 (1.0)	7 (1.0)
Cancer	119 (8.4)	48 (7.3)	71 (9.3)
Crohn’s	33 (2.3)	7 (0.1)	26 (3.4)
Epilepsy/Seizure Disorder	69 (4.6)	31 (4.7)	38 (5.0)
Glaucoma	21 (1.5)	20 (3.0)	1 (0.1)
HIV	11 (< 0.1)	9 (1.4)	2 (0.1)
Intractable Pain	711 (50)	305 (46.4)	406 (53.1)
Multiple Sclerosis	17 (<0.1)	5 (0.7)	12 (1.6)
Muscular Dystrophy	4 (<0.1)	3 (0.4)	1 (0.1)
Parkinson’s disease	17 (<0.1)	15 (2.2)	2 (0.1)
PTSD	218 (15.3)	124 (18.9)	94 (12.3)
Severe Muscle Spasm	163 (11.4)	69 (10.5)	94 (12.3)
Spasticity	20 (<0.1)	12 (1.8)	8 (1.0)
More than one indication	195 (13.7)	51 (7.7)	144 (18.8)

**Table 5:** Comparison of MM recommendations for male and female patients in Louisiana MM dispensaries.

Doses	Total	Male	Female
Recommendation, N (%)	1195	598 (50)	597 (50)
**THC-Rich**	**625 (52.3)**	**321 (53.7)**	**304 (50.9)**
10 mg THC-Rich	87 (7.2)	43 (7.2)	44 (7.4)
7.5 mg THC-Rich	2 (0.1)	2 (0.3)	0 (0)
5.0 mg THC-Rich	244 (20.4)	125 (21)	119 (20)
2.5 mg THC-Rich	285 (23.8)	150 (25)	135 (23)
1.5 mg THC-Rich	7 (0.6)	1 (0.2)	6 (1.0)
**Balanced**	**495 (41.4)**	**242 (40.4)**	**253 (42.3)**
10 mg Balanced	3 (0.2)	1 (0.2)	2 (0.3)
7.5 mg Balanced	1 (0.1)	1 (0.2)	0 (0)
5.0 mg Balanced	146 (12.2)	75 (12.5)	71 (11.9)
2.5 mg Balanced	229 (19.1)	113 (18.9)	116 (19)
1.25 mg Balanced	116 (9.7)	52 (8.7)	64 (11)
**CBD-Rich**	**75 (6.3)**	**35 (5.8)**	**40 (6.7)**
20 mg CBD-Rich	4 (0.2)	0 (0)	4 (0.7)
10 mg CBD-Rich	9 (0.8)	5 (0.6)	4 (0.7)
5 mg CBD-Rich	9 (0.8)	3 (0.3)	6 (1.0)
2.5 mg CBD-Rich	17 (1.4)	11 (1.8)	6 (1.0)
1.25 mg CBD-Rich	36 (3.0)	16 (2.7)	20 (3.4)
